# Effect of N6-Methyladenosine Regulators on Progression and Prognosis of Triple-Negative Breast Cancer

**DOI:** 10.3389/fgene.2020.580036

**Published:** 2021-01-28

**Authors:** Shanshan Wang, Xuan Zou, Yajie Chen, William C. Cho, Xiang Zhou

**Affiliations:** ^1^Cancer Institute, Fudan University Shanghai Cancer Center, Fudan University, Shanghai, China; ^2^Department of Oncology, Shanghai Medical College, Fudan University, Shanghai, China; ^3^Department of Medical Oncology, Fudan University Shanghai Cancer Center, Shanghai, China; ^4^Department of Radiation Oncology, Fudan University Shanghai Cancer Center, Fudan University, Shanghai, China; ^5^Department of Clinical Oncology, Queen Elizabeth Hospital, Hong Kong, China; ^6^Institutes of Biomedical Sciences, Fudan University, Shanghai, China; ^7^Key Laboratory of Breast Cancer in Shanghai, Fudan University Shanghai Cancer Center, Fudan University, Shanghai, China

**Keywords:** TNBC, N6-methyladenosine, nomogram, progression, prognosis

## Abstract

**Background:** The N6-methyladenosine (m^6^A) modification plays a critical role in cancer development. Little is known about the m^6^A modification in triple-negative breast cancer (TNBC), the most aggressive subtype of breast cancer. Thus, the prognostic value of m^6^A RNA methylation in TNBC deserves exploration.

**Methods:** The expression levels of the 13 m^6^A methylation regulators were compared between the 98 TNBC tumor samples and normal tissue samples based on the transcriptome profiles from The Cancer Genome Atlas (TCGA). The association between the m^6^A regulators and patients' overall survival was assessed by Kaplan-Meier survival analysis and Cox regression analysis. Lasso regression analysis was conducted to construct a prognostic model based on the m^6^A methylation system. The prognostic performance of the identified model was validated in GSE88847 and GSE135565 datasets. A nomogram combining the TNM stage and the m^6^A prognostic model was further constructed for the survival prediction of TNBC patients.

**Results:** The m^6^A regulator genes were remarkably dysregulated in TNBC tumor tissues, with *ALKBH5, YTHDF2, HNRNPC, KIAA1429*, and *RBM15* significantly up-regulated and *FTO, YTHDC1, YTHDC2, METTL3, METTL14*, and *ZC3H13* significantly down-regulated (*P* < 0.01). The expression level of *ALKBH5* was an independent unfavorable prognostic factor (*HR* = 3.327, *P* = 0.006), while *METTL14* (*HR* = 0.425, *P* = 0.009) was an independent favorable prognostic factor for TNBC patients. A prognostic model consisting of *ALKBH5* and *METTL14* was therefore proposed displaying higher accuracy of risk prediction when combined with TNM stage with an AUC of 0.791. The prognostic value of the identified signature remained consistent within the two external validation datasets.

**Conclusion:** The m^6^A methylation regulators were significantly dysregulated in TNBC tissues and could constitute a novel prognostic signature for the survival prediction of TNBC patients.

## Introduction

Breast cancer (BC) is one of the most life-threatening malignancies in females. According to the cancer epidemiological investigation of the American Cancer Society, BC is the most common malignancy and the second leading cause of cancer-related death among women in the United States in 2020 (Siegel et al., 2020). Globally, female BC remains a heavy heath burden in the great majority of regions, including both developing and developed countries (Bray et al., 2018). Based on the heterogeneity of gene expression profiles (i.e., estrogen receptor [ER], progesterone receptor [PR], and human epidermal growth factor receptor 2 [HER2]), BC is typically classified into four main molecular subtypes: luminal A (ER and/or PR-positive, HER2-negative), luminal B (ER and/or PR-positive, HER2-positive), HER2-enriched (ER and PR-negative, HER2-positive), and triple-negative (ER, PR, and HER2-negative) (Carey et al., 2006). The different subtypes of BC present varied characteristics with regard to biological properties, treatment tactics, and clinical outcomes (Di Cosimo and Baselga, 2010; Lu, 2018). Since triple-negative breast cancer (TNBC) tends to behave more aggressively and has a relatively worse prognosis than the other subtypes, the appropriate prognostic prediction strategy of TNBC is considered to have critical importance in disease management (Bianchini et al., 2016; Bao et al., 2019). However, apart from the traditional TNM staging evaluation system, other prognostic biomarkers sporadically proposed by limited sample surveys are likewise deficient in stability and consistency of survival prediction for TNBC (Park et al., 2011).

A growing number of studies have demonstrated the role of misregulated RNA modifications in cancer development. Up to now, more than one hundred types of RNA modifications have been discovered, which gives rise to a new frontier in cancer research apart from well-studied DNA or protein modifications (Barbieri and Kouzarides, 2020). N6-methyladenosine (m^6^A), which refers to the methylation of adenosine at nitrogen-6 position, is the most common and best characterized RNA modification found in both coding and non-coding RNAs (Pan, 2013). It has been well-documented that the m^6^A modification is regulated by the dynamic interplay between the “writer,” “reader,” and “eraser” proteins (Dai et al., 2018; Reichel et al., 2019). Briefly, the m^6^A methylation process is catalyzed by “writers”—the methyltransferases including *METTL3, METTL14, WTAP, KIAA1429, RBM15*, and *ZC3H13*, and reversed by “erasers”—the demethylases including *FTO* and *ALKBH5*. The m^6^A binding proteins (*YTHDC1, YTHDC2, YTHDF1, YTHDF2*, and *HNRNPC*) function as “readers” which recognize specific m^6^A modified RNAs and mediate post-transcriptional regulation (Yang et al., 2018). These m^6^A methylation regulators are frequently dysregulated in cancer and may profoundly influence tumor initiation, progression, and metastasis (Lan et al., 2019). Therefore, the expression profile of the 13 m^6^A regulator genes in tumor tissues and its potential as prognostic biomarkers for cancer survival is worth studying.

Thus, far, evidence regarding the role of m^6^A methylation in TNBC is still quite limited. Moreover, whether the m^6^A regulatory system can raise a novel signature for the prediction of TNBC survival remains largely unknown. To address these issues, the present study mainly explored the expression pattern of the m^6^A regulatory genes in TNBC tissues and its correlation with TNBC prognosis based on the data of The Cancer Genome Atlas (TCGA) and Gene Expression Omnibus (GEO).

## Methods

### Data Source and Study Design

The 98 TNBC tumor tissue samples in The Cancer Genome Atlas Breast Invasive Carcinoma (TCGA-BRCA) dataset were trained for the construction of prognostic model and nomogram. The follow-up time and clinicopathologic parameters of the 98 TNBC patients as well as the level 3 transcriptome profiling data of tumor tissues and the 114 normal breast tissues were downloaded from https://portal.gdc.cancer.gov/. The Fragments Per Kilobase per Million (FPKM)-normalized gene expression data was obtained and normalized by “limma” package. The 13 m^6^A regulator genes were annotated according to the human reference genome assembly GRCh38, and the gene expression was normalized using the limma package and combined into a matrix (Ritchie et al., 2015).

The TNBC samples of two microarray datasets of gene expression profiling—GSE88847 and GSE135565 were tested to confirm the prognostic value of the identified signature. GSE88847 recorded the metastasis, recurrence, and survival status of 37 TNBC patients. GSE135565 contained the TNM stage, standardized uptake value (SUV), and survival status information of 100 TNBC patients. The corresponding series matrix files and platform files were obtained from GEO (https://www.ncbi.nlm.nih.gov/). Probe IDs of the arrays were transformed into gene symbols according to the corresponding platform information. For each dataset, the expression levels of the 13 m^6^A regulator genes and the clinical information of patient samples were merged into a matrix for further analysis.

All data used in the study was obtained from TCGA, and thus ethical approval and informed consent were not required.

### Construction of the Prognostic Model

To enhance the accuracy of survival prediction, Lasso (least absolute shrinkage and selection) regression analysis using the glmnet (Friedman et al., 2010) and caret packages (https://CRAN.R-project.org/package=caret) was performed for variable selection. The coefficient of each variable in the regression model was recorded and used to calculate the risk score of each patient. The patient samples of the TCGA and two GEO datasets were separately divided into high-risk and low-risk groups according to the median value of the estimated risk scores.

### External Validation of the Model

In GSE88847 (*n* = 37 TNBC samples), the calculated risk scores were compared between patients presenting different disease characteristics (i.e., no-metastasis vs. metastasis, no-recurrence vs. recurrence, and alive *vs*. dead) to verify whether patients with advanced disease had higher risk scores. In GSE135565 (*n* = 84 TNBC samples), subgroup analysis was conducted to evaluate the correlation between risk score and TNM stage and SUV. The log-rank test was used to compare the survival distributions of high-risk and low-risk patients.

### Development and Evaluation of a Nomogram

In the TCGA dataset, the calculated risk score and TNM stage was integrated into a nomogram for the more precise prediction of TNBC prognosis. The accuracy and clinical utility of the predictive system was assessed by calibration curve analysis and decision curve analysis (DCA), respectively.

### Gene Set Enrichment Analysis (GSEA)

The pathways potentially involved by the high-risk and low-risk TNBC patients were explored by gene set enrichment analysis (GSEA). The classical gene sets of Kyoto Encyclopedia of Genes and Genomes (KEGG) and Gene Oncology (GO) assemblies were analyzed. For each gene set, normalized enrichment score (NES) and adjusted *P*-value was calculated by comparing the comprehensive transcriptome expression data between the high-risk and low-risk subgroups. A normalized *P* < 0.05 was considered to be significant.

### Statistics Methods and Tools

The Mann–Whitney *U-*test was performed to compare the gene expression difference between TNBC tumor tissues and normal tissues as well as the risk scores of patients with varied clinicopathologic features. The univariate and multivariate Cox regression analysis was conducted to select prognosis-associated genes. The Kaplan-Meier (KM) curve and log-rank test was used to make survival comparison between two subgroups. Receiver operating characteristic (ROC) curves were developed to illustrate the prognostic value of the identified signature. In this study, the rate of type I error was set as 0.05. When selecting the differentially expressed genes and the significantly involved pathways, the false discovery rate (FDR) was calculated to adjust the initial *P*-values, and the cutoff of FDR was set as 0.05. Statistical analysis in this study was conducted using the R software (version 3.6.3).

## Results

### Expression of the m^6^A RNA Methylation Regulators in TNBC

**Figure 1:**
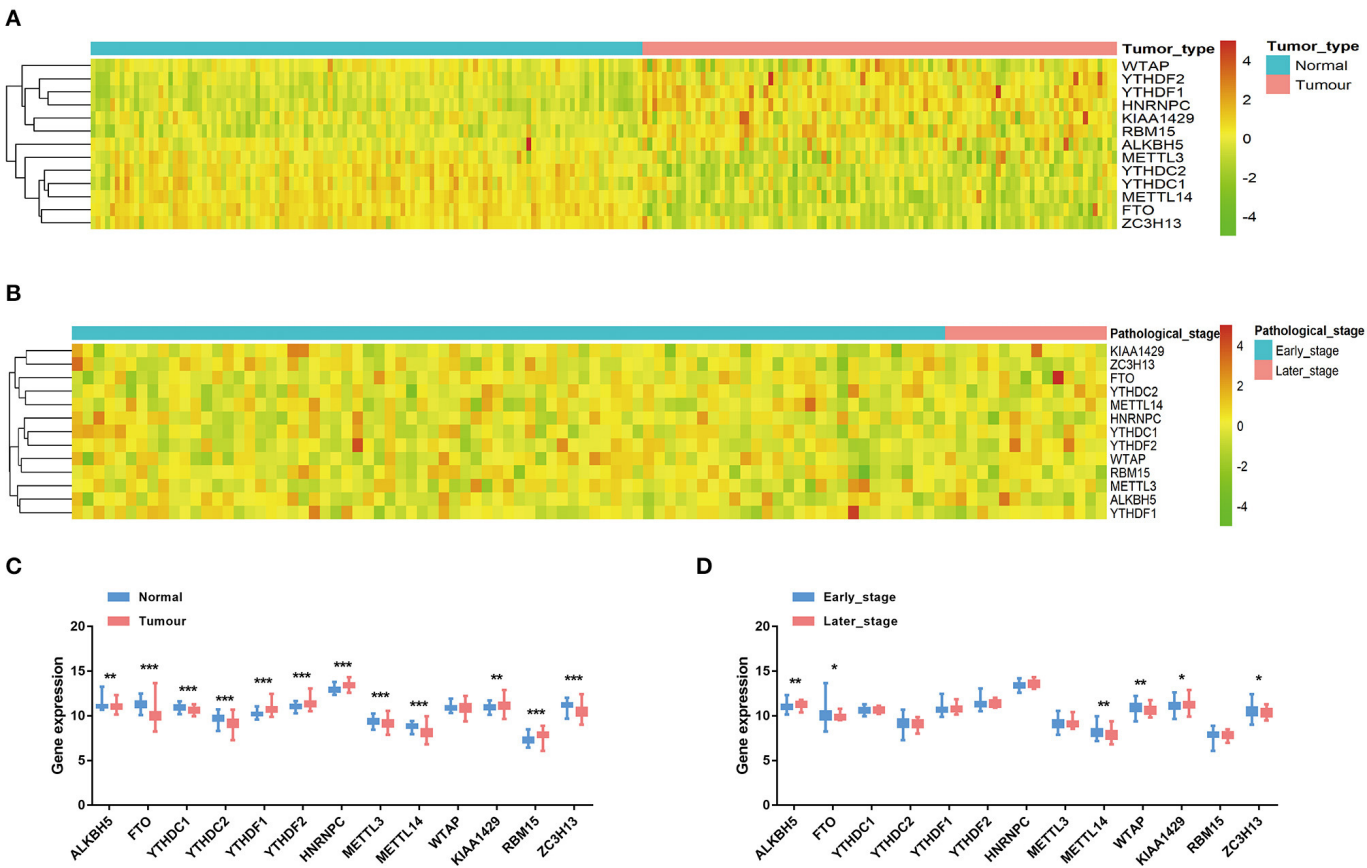
The expression of the 13 m^6^A RNA methylation regulators in TNBC. **(A)** The heatmap of gene expression in TNBC tissues (*n* = 98) and normal tissues (*n* = 114). **(B)** The heatmap of gene expression in TNBC patients with different pathological stages. **(C)** The boxplot of gene expression levels in TNBC tissues and normal tissues. Horizontal line: mean with 95% confidence interval (CI). **(D)** The boxplot of gene expression levels in early-stage (stage I and stage II) TNBC patients and late-stage (stage III and stage IV) TNBC patients. Horizontal line: mean with 95% CI. *P < 0.05, **P < 0.01, ***P < 0.001.

The mRNA expression levels of the 13 m^6^A methylation regulators were analyzed in the TCGA-BRCA dataset. Except *WTAP*, the abnormal expression of all the other 12 genes was observed in the 98 TNBC tumor tissues compared with the 114 normal tissues (Figures 1A,C). Specifically, *ALKBH5, YTHDF2, HNRNPC, KIAA1429*, and *RBM15* were significantly up-regulated in TNBC tissues, while *FTO, YTHDC1, YTHDC2, METTL3, METTL14*, and *ZC3H13* were significantly down-regulated (*P* < 0.01). In addition, the expression of the 13 genes was compared between the early-stage (stage I and stage II) and late-stage (stage III and stage IV) TNBC patients (Figures 1B,D). The significant up-regulation of *ALKBH5* and *KIAA1429*, and down-regulation of *FTO, METTL14, WTAP*, and *ZC3H13* could be observed in the TNBC patients with advanced stages (*P* < 0.05), indicating that these m^6^A regulators may be associated with progression of TNBC.

### Prognostic Value of the m^6^A RNA Methylation Regulators for TNBC

**Figure 2:**
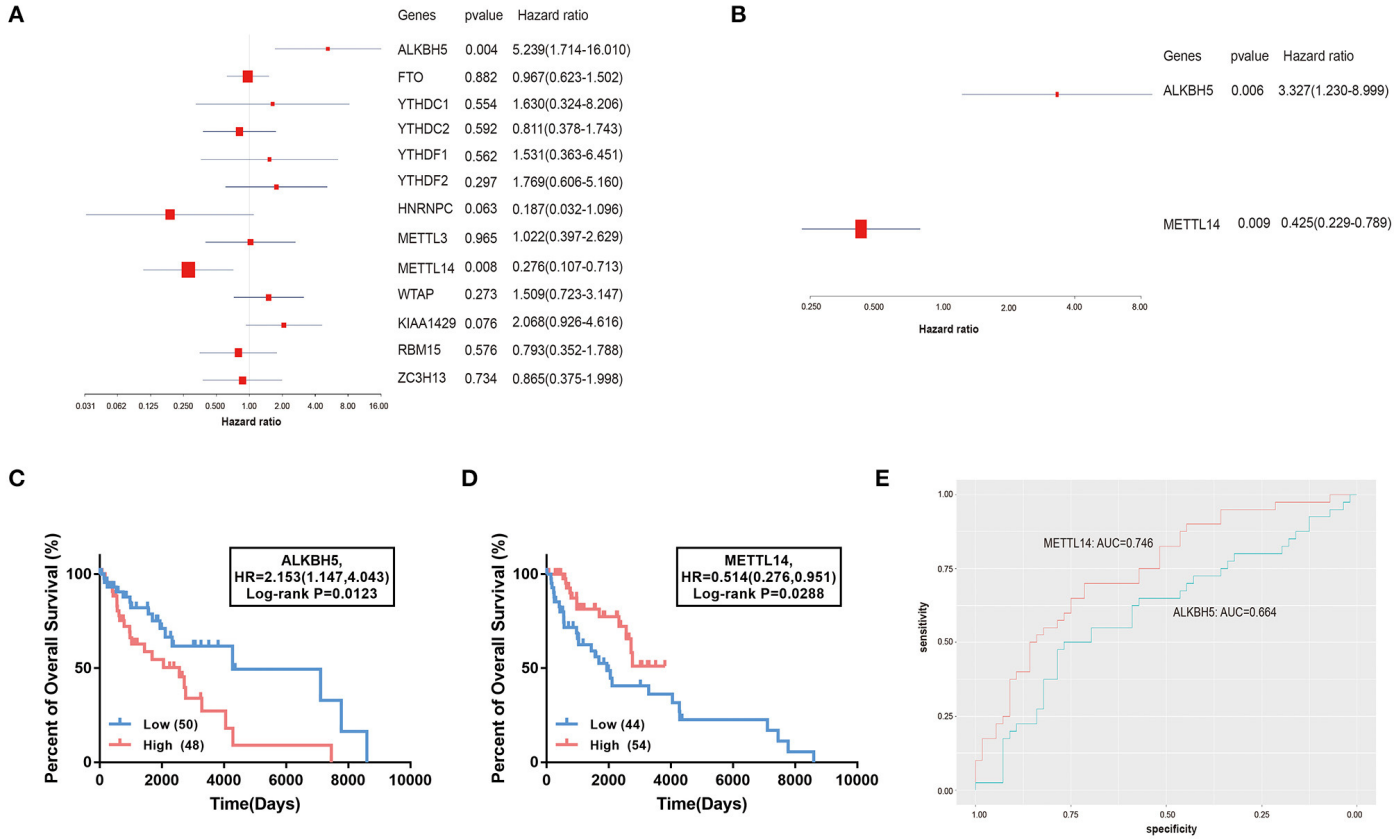
Association of the 13 m^6^A RNA methylation regulators with the overall survival of TNBC patients. **(A,B)** The forest plot of univariate and multivariate Cox regression analysis. **(C,D)** The comparison of survival curves between high- and low- *ALKBH5* and *METTL14* expression subgroups. HR, hazard ratio. **(E)** The ROC curves of the two genes suggesting the sensitivity and specificity for survival prediction. ROC, receiver operating characteristic; AUC, area under the curve.

The association between the 13 genes and the clinical outcome of TNBC patients was explored in the TCGA-BRCA dataset. The univariate Cox regression analysis suggested that the increased expression of *ALKBH5* and decreased expression of *METTL14* is significantly correlated with the poor prognosis of TNBC patients (Figure 2A). The multivariate Cox regression analysis further confirmed that the expression level of *ALKBH5* is an independent unfavorable prognostic factor (*HR* = 3.327, 95% confidence interval [CI]: 1.230–8.999, *P* = 0.006), and *METTL14* (*HR* = 0.425, 95% CI: 0.229–0.789, *P* = 0.009) is an independent favorable prognostic factor for TNBC patients (Figure 2B). The log-rank survival analysis showed that the TNBC patients with higher expression of *ALKBH5* or lower level of *METTL14* have remarkable poorer survival (*P* < 0.05; Figures 2C,D). For the prognostic prediction of TNBC, the AUCs of *ALKBH5* and *METTL14* were 0.746 and 0.664, respectively (Figure 2E), authenticating the effectiveness of the two potential prognostic factors.

### Building of a Survival Prediction Model

**Figure 3:**
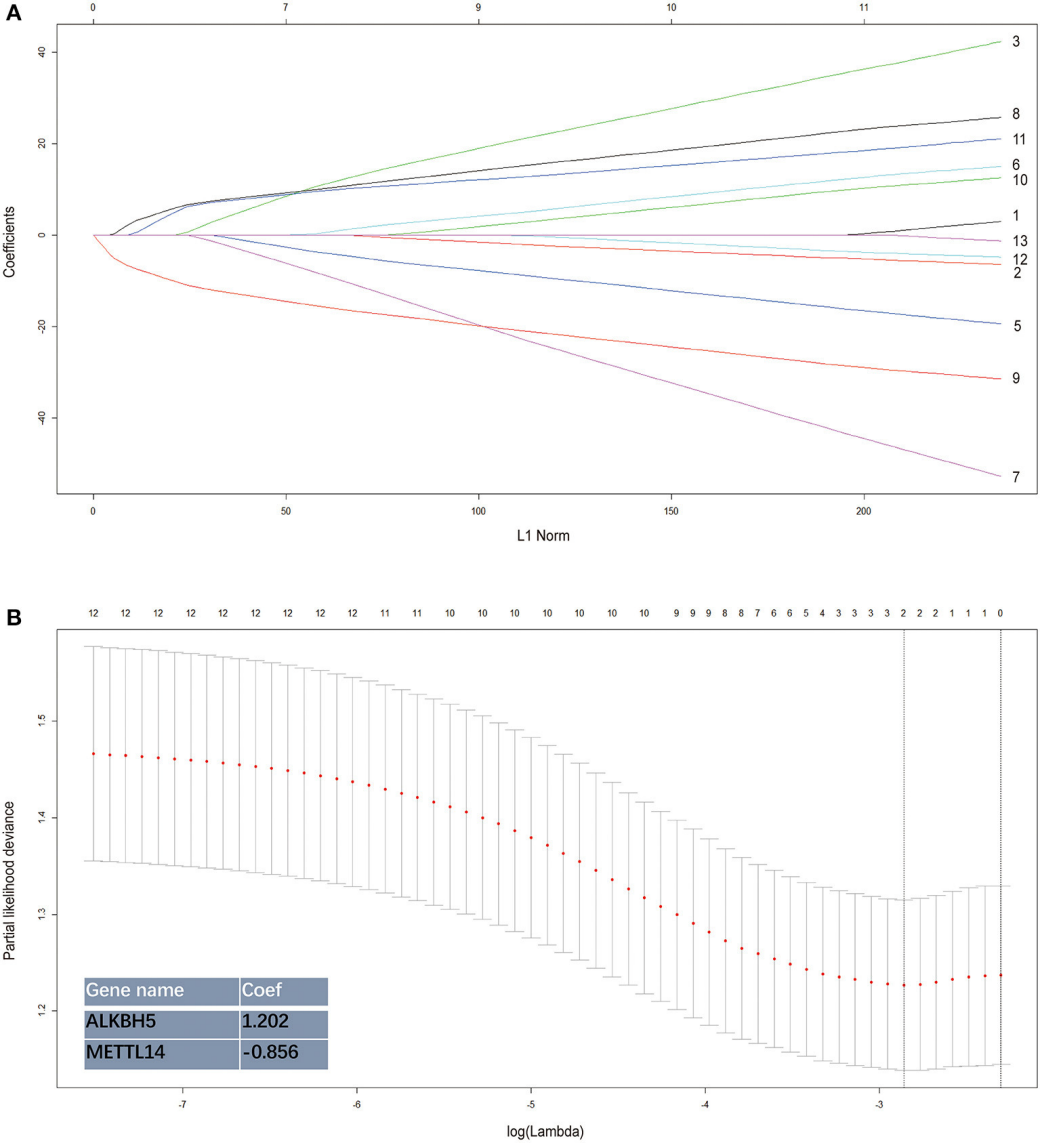
Establishment of the risk signature integrating the 2 m^6^A RNA methylation regulators. The coefficients estimated by the Lasso regression method are presented. Each curve in the **(A)** represents the path of a lasso coefficient against the L1-norm (the penalty term for lasso) when λ changes. A coefficient that becomes non-zero when λ changes enters the LASSO regression model. **(B)** The coefficients estimated by the Lasso regression method are presented.

**Figure 4:**
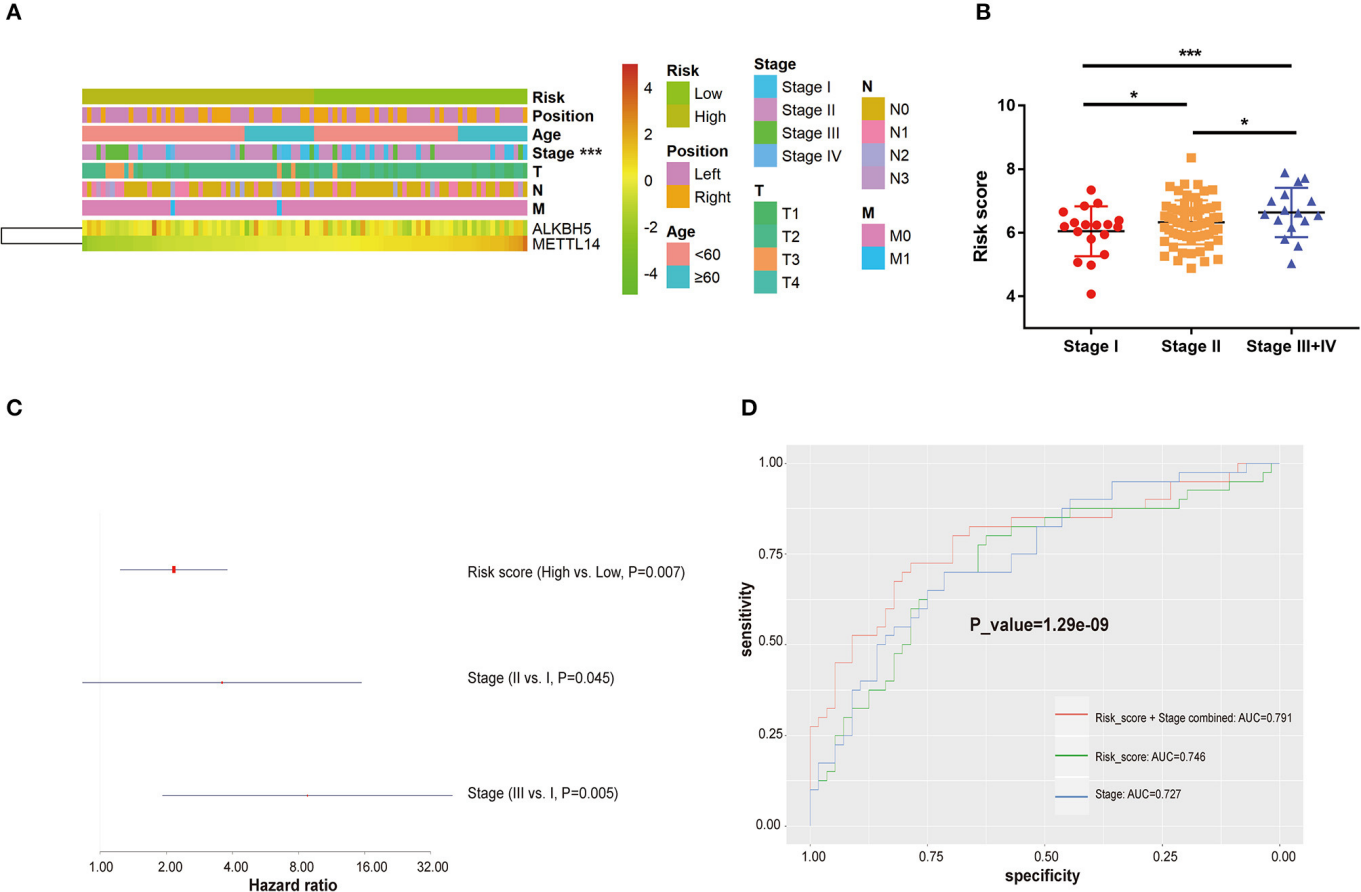
The relation between risk score and clinicopathological characteristics in TNBC. **(A)** The heatmap of risk distribution in TNBC patients with varied clinicopathological characteristics. **(B)** The comparison of risk score among patients with different TNM stages (Stage I+II vs. Stage III+IV). **(C)** Multivariate Cox regression analysis of the association between clinicopathological factors (risk score and stage) and overall survival of patients. **(D)** ROC curves comparing predictive sensitivity and specificity between the risk score and stage system. ROC: receiver operating characteristic. AUC, area under the curve. *P < 0.05, ***P < 0.001.

The two prognostic factors were combined using the Lasso regression method to build a prognostic model for the survival prediction of TNBC (Figure 3). The risk score of each TNBC patient estimated by the model could be calculated by the formula “1.202 × *ALKBH5* expression−0.856 × *METTL14* expression.” In the TCGA-BRCA dataset, the 98 TNBC patients were separated into the high-risk and low-risk groups based on the median value of the calculated risk scores. The heatmap of risk distribution among the 98 patients with different clinicopathologic features was shown in Figure 4A.

In the TCGA cohort, the risk score was significantly associated with TNM stages. The TNBC patients in the late stage (Stage III/IV) had higher risk scores than those in Stage I and Stage II (*P* < 0.05; Figure 4B). The multivariate Cox regression analysis that combined the risk score and TNM stage was performed to assess the independent predictive value of the model. As shown in Figure 4C, in the TCGA dataset, the estimated risk score could predict the clinical outcomes of TNBC patients independently of TNM stage (HR > 1, *P* = 0.007). The AUCs of the model to predict TNBC survival was 0.746, higher than that of the TNM staging system (AUC = 0.727). When the two parameters of the risk score and TNM stage were combined, the predictive performance was further improved with an AUC of 0.791 (Figure 4D).

### External Validation in GEO Datasets

**Figure 5:**
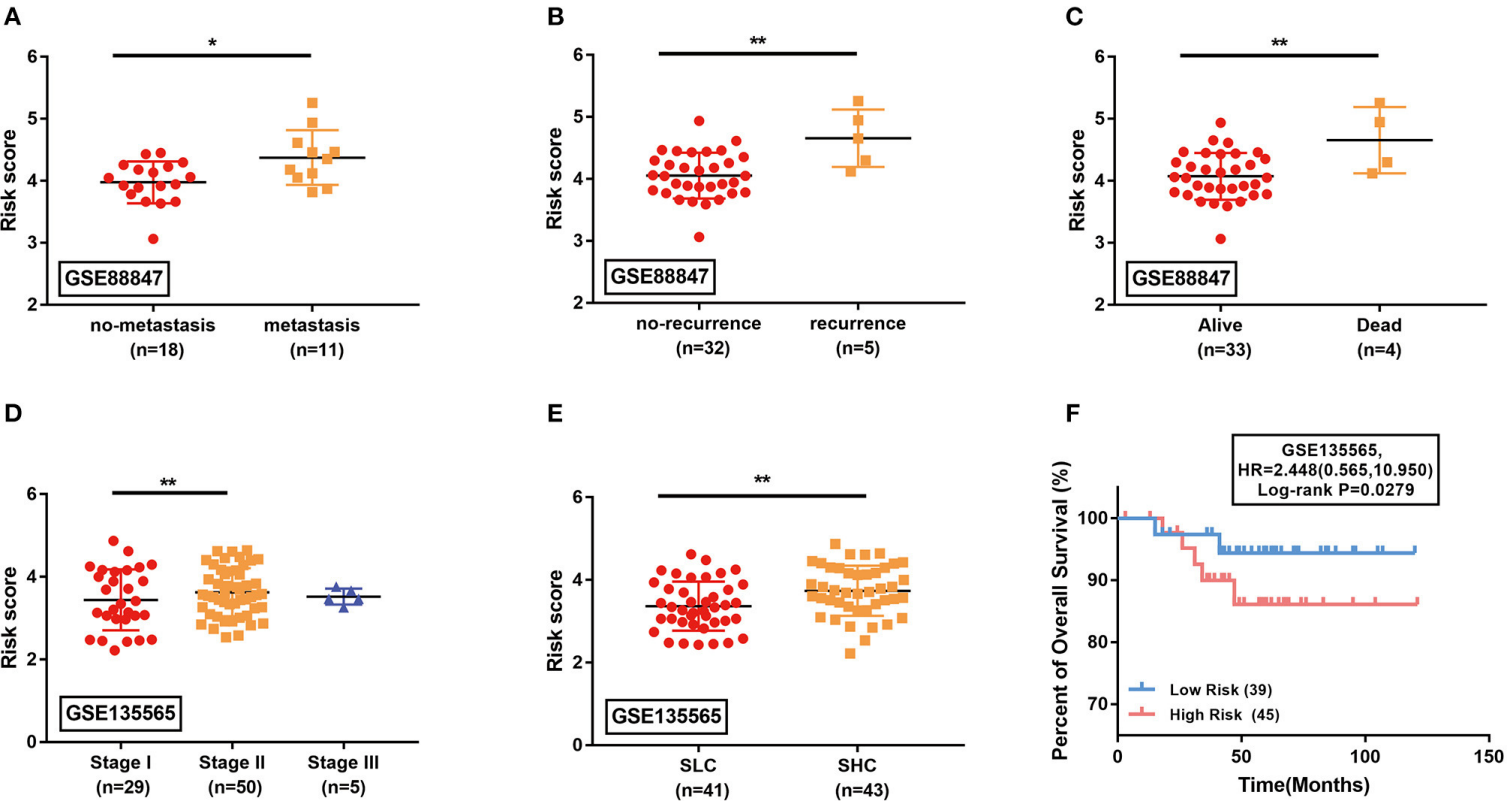
External validation of the identified signature in GSE88847 **(A–C)** and GSE135565 **(D–F)** datasets. **(A–E)** Comparison of the estimated risk scores in different subgroups. Horizontal line: mean with SD. SLC, standardized uptake value (SUV)-low cluster; SHC, SUV-high cluster. *P < 0.05, **P < 0.01. **(F)** The Kaplan-Meier survival curves comparing the overall survival between high-risk and low-risk TNBC patients in GSE135565. HR, hazard ratio.

The GSE88847 and GSE135565 datasets that contained the transcriptome profiling data of TNBC patients were analyzed to validate the prognostic value of the identified model. In GSE88847, the results showed that the metastatic (Figure 5A), recurrent (Figure 5B), and dead cases (Figure 5C) had significant higher risk scores than the controls (*P* < 0.05). In GSE135565, the association between risk scores and TNM stage as well as standardized uptake value (SUV) was evaluated. The risk scores of the TNBC patients in Stage II were much higher than those in Stage I (*P* < 0.01; Figure 5D). The SUV-high cluster (SHC) presented significant higher risk scores than the SUV-low cluster (SLC; *P* < 0.01; Figure 5E). The overall survival of patients in the GSE135565 dataset was further calculated. The log-rank survival analysis verified that the estimated high-risk patients exactly have a poorer prognosis than the low-risk patients (Figure 5F).

### A Nomogram to Estimate Prognosis in TNBC

**Figure 6:**
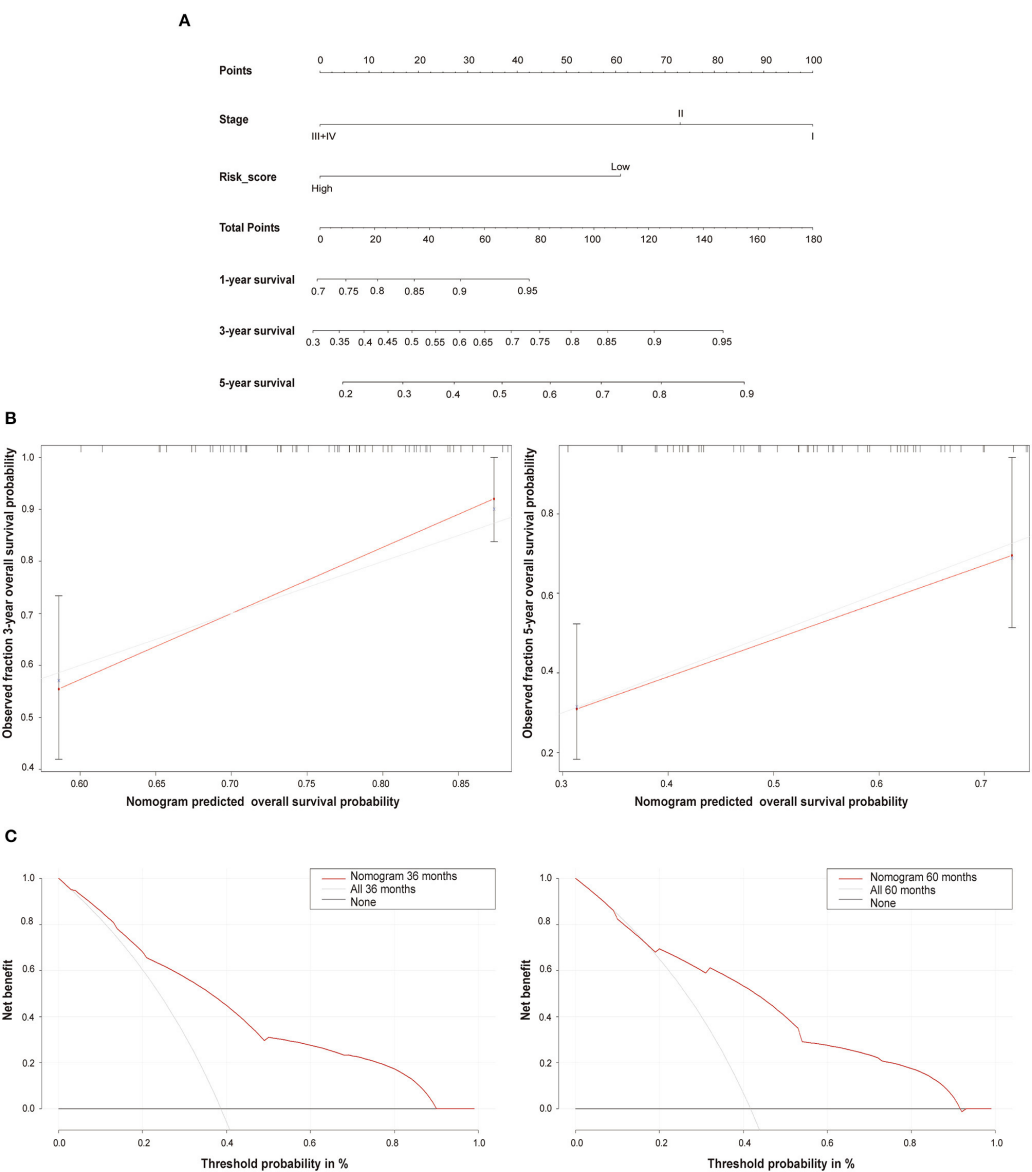
The nomogram prediction model. **(A)** The nomogram combining the risk score and TNM stage system for the survival prediction of TNBC. **(B)** The calibration curves comparing the estimated 3- and 5-years survival probability with the actual survival probability of TNBC patients. **(C)** The 3- and 5-years decision curve analysis (DCA) evaluating the clinical utility of the nomogram.

A nomogram integrating the risk score and TNM stage was built to estimate the prognosis of TNBC patients using the TCGA dataset (Figure 6A). The calibration curves indicated that the nomogram can estimate 3- and 5-years survival probability of TNBC patients with high accuracy (Figure 6B). The DCA curve analysis further demonstrated the clinical utility of the nomogram (Figure 6C).

### Functional Enrichment Analysis

**Figure 7:**
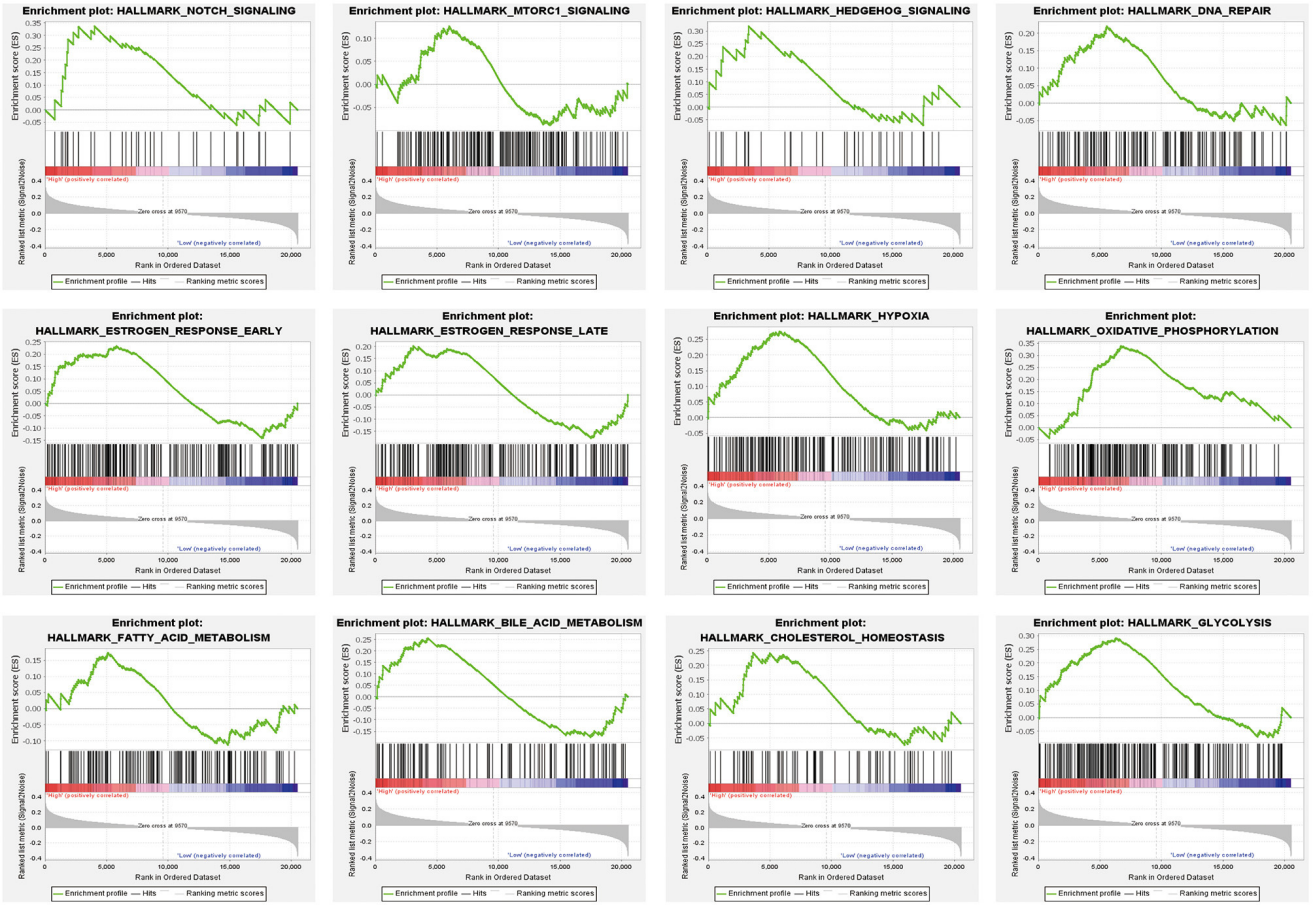
Significant pathways that may be associated with the estimated high-risk patients compared with the low-risk group using gene set enrichment analysis (GSEA).

Finally, we explored the significant pathways potentially involved by the high-risk patients compared with the low-risk (*P* < 0.05) (Figure 7). Some well-appreciated cancer-related pathways were identified, such as Notch, mTOR, and Hedgehog signaling pathways. Also, several critical biological processes including DNA repair, hypoxia, and glucose and lipid metabolism that play important roles in cancer cell survival, growth, chemoresistance, and angiogenesis were highly associated with the estimated high-risk patients and, probably, m^6^A-associated TNBC progression.

## Discussion

TNBC is characterized by the negative expression of ER, PR, and HER2, and accounts for around 15–20% of all diagnosed breast cancer cases (Lee et al., 2009). Unlike the other subtypes, TNBC is more common in younger female patients and usually indicates poorer clinical outcomes despite intensive treatment (Gnerlich et al., 2009). Clinically, TNBC tends to behave more aggressively than the other subtypes, with a higher risk of recurrence, metastasis, and cancer-related death (Mustacchi and De Laurentiis, 2015). At present, it remains a challenge to improve the overall survival of TNBC patients owing to the absence of efficient treatment strategies, which makes the proposal of appropriate prognostic models necessary for risk prevention. Previous studies have identified several traditional clinic-pathological features (i.e., age, tumor size, and nodal status) in addition to some well-recognized prognostic factors, such as androgen receptor expression, Ki67 index and basal cytokeratin status, to provide reference for the outcome estimation of TNBC patients (Rakha et al., 2007; Kashiwagi et al., 2011; Lai et al., 2011; Adamo et al., 2017). However, a prediction system based only on several anatomy features or molecular properties may be unstable to reflect the overall and long-term changes in TNBC (Colozza et al., 2005). A comprehensive model brought up from a new angle of view is thus in urgent need to provide higher predictive accuracy for risk stratification in TNBC.

In the present study, we focused on the m^6^A modification system and constructed a comprehensive model for the prognostic prediction of TNBC. The m^6^A modifications exert comprehensive functions by participating in a broad range of post-transcriptional regulation processes, such as RNA transcription, translation, splicing, and degradation. Through altering the expression of target genes, m^6^A methylation exerts deep influence on the corresponding cellular events and cell fate (Chen et al., 2019; He et al., 2019). In various cancer types, the abnormal m^6^A modification has been gradually appreciated, attracting increasing attention to the investigation of m^6^A function in cancer biological processes. For example, the m^6^A methyltransferases METTL3/14 and WTAP could act as oncogenic factors by promoting the translation of several oncogenes including c-*MYC, BCL2, PTEN*, and *mTOR* in acute myeloid leukemia (Bansal et al., 2014; Vu et al., 2017; Sorci et al., 2018; Weng et al., 2018). The tumor-promoting effect of METTL3 was also reflected by its activity to mediate YTHDF2-dependent mRNA decay of SOCS2, a suppressor of cancer metastasis (Chen et al., 2018). Interestingly, METTL14 was found to be positively associated with cancer prognosis in several other scenarios. For instance, this protein could suppress metastasis of hepatocellular carcinoma by modulating m^6^A-dependent microRNA processing and biogenesis (Ma et al., 2017). Wang et al. also identified METTL14 as an independent favorable prognostic marker in clear cell renal cell carcinoma (Wang et al., 2020). In breast cancer, the tumor-promoting activity of the m^6^A regulators such as METTL3 and ALKBH5 were also described (Zhang et al., 2016; Cai et al., 2018). However, the role of m^6^A regulators in progression and prognosis of TNBC remains largely elusive.

In this study, we unveiled that the m^6^A methylation regulators are significantly dysregulated in TNBC tumor tissues compared with normal tissues based on the TCGA transcriptome profiling data (Figure 1). Notably, up-regulation of ALKBH5 and down-regulation of METTL14 could independently indicate an adverse prognosis in TNBC patients (Figure 2). The findings were consistent with the previous studies. Wu et al. showed that reduction of the m^6^A level by decreasing METTL14 or elevating ALKBH5 expression promotes proliferation and migration of breast cancer cells (Wu et al., 2019). Zhang et al. revealed that overexpression of ALKBH5 enhances NANOG expression by mediating m^6^A-demethylation of its mRNA, consequently leading to the stem-like features of breast cancer cells (Zhang et al., 2016). The oncogenic role of ALKBH5 was also reported in glioblastoma (Zhang et al., 2017). However, METTL14 was found to act as both the tumor suppressor and the oncogenic protein by collaborating with different m^6^A “reader” proteins in the context of diverse cancer types (Cui et al., 2017; Ma et al., 2017; Liu et al., 2018; Weng et al., 2018). Therefore, our findings along with the above mentioned studies further demonstrate the important roles of the 2 m^6^A modulators in TNBC progression and prognosis.

By performing Lasso regression analysis, we constructed a prognostic model incorporating *METTL14* and *ALKBH5* expression for the survival prediction of TNBC (Figure 3). TNBC patients were divided into the high-risk and low-risk groups based on the estimated risk scores. Subgroup analyses conducted in the TCGA and external validation GEO datasets suggested that patients with more advanced disease status have significant higher risk according to this estimating system. When the TNM stage system and the Lasso regression model were combined, the prognostic performance was further improved with an AUC of 0.791 (Figure 4). Hence, we further integrated the two parameters together and built a comprehensive nomogram to estimate the survival probability of TNBC patients (Figures 5, 6). The calibration curves and DCA curves showed the clinical significance of this novel prognostic signature.

This study described the first association of m^6^A modification with prognostic evaluation system in TNBC. Compared with the classical TNM stage, the identified signature had better performance in the survival prediction of TNBC patients. Nevertheless, it is intriguing to mechanistically investigate the role of ALKBH5 and METTL14 in TNBC, which would be rewarding for future development of more accurate prognostic models and eventually therapeutic approaches.

## Conclusion

In summary, the present study has demonstrated for the first time the aberrant expression of the m^6^A regulators in TNBC tissues. A novel prognostic model incorporating the expression of *ALKBH5* and *METTL14* was constructed to estimate the risk of TNBC patients.

## Data Availability Statement

The datasets presented in this study can be found in online repositories. The names of the repository/repositories and accession number(s) can be found in the article.

## Ethics Statement

All data used in the study was obtained from TCGA, and thus ethical approval and informed consent were not required.

## Author Contributions

SW, XZo, and YC conducted data mining, analysis, and drafted the manuscript. WC provided critical instructions. XZh designed, supervised the study, and composed the manuscript. All authors contributed to the article and approved the submitted version.

## Conflict of Interest

The authors declare that the research was conducted in the absence of any commercial or financial relationships that could be construed as a potential conflict of interest.
